# Large-scale organellar genomics reveals maternal evolutionary history and dynamism in the wolfberries

**DOI:** 10.3389/fpls.2026.1956613

**Published:** 2026-10-27

**Authors:** Yue Yin, Luyao Wang, Wei An, Xiaorong Bai, Xiyan Zhang, Youlong Cao, Penghao Wang, Jun He, Xiaoya Qin, Yajun Wang, Zhibing Li, Zhigang Shi, Luke R. Tembrock, Junhu Kan

**Affiliations:** 1 Key Laboratory of National Forestry and Grassland Administration on Goji, Research of Institute of Wolfberry Science, NingXia Academy of Agriculture and Forestry Sciences, Yinchuan, China; 2 State Key Laboratory of Tropical Crop Breeding, Shenzhen Branch, Guangdong Laboratory of Lingnan Modern Agriculture, Key Laboratory of Synthetic Biology, Ministry of Agriculture and Rural Affairs, Agricultural Genomics Institute at Shenzhen, Chinese Academy of Agricultural Sciences, Shenzhen, China; 3 Shanxi Key Laboratory of Germplasm Resources Innovation and Utilization of Vegetable and Flower, College of Horticulture, Shanxi Agricultural University, Taiyuan, Shanxi, China; 4 School of Medical, Molecular and Forensic Sciences, Murdoch University, Perth, WA, Australia; 5 Western Crop Genetics Alliance, Food Futures Institute and School of Agriculture, Murdoch University, Perth, WA, Australia; 6 Cooper Medical School of Rowan University, Camden, NJ, United States; 7 College of Landscape Architecture, Zhejiang A&F University, Hangzhou, China; 8 Department of Agricultural Biology, College of Agricultural Sciences, Colorado State University, Fort Collins, CO, United States

**Keywords:** large-scale organellar genomics, maternal inheritance, mitogenome dynamism, structural variation, wolfberry

## Abstract

**Introduction:**

The organellar genomic diversity and maternal evolutionary history of the *Lycium* complex remain incompletely understood.

**Methods:**

We compared organellar genomes from 201 accessions, with dense sampling of *L. barbarum* and *L. ruthenicum* plus representative relatives. A single *L. ruthenicum* mitogenome was assembled de novo and used as the reference for comparisons of 186 LR and LB-complex accessions.

**Results:**

Plastome analyses resolved seven maternal genetic clusters and identified patterns consistent with complex maternal ancestry within the red wolfberry complex. However, plastid data alone cannot distinguish hybridization, introgression, and incomplete lineage sorting. All 201 plastomes retained a conserved quadripartite structure and 79 unique protein-coding genes. Illumina depth profiles identified a candidate 5.5-kb structural difference with strongly reduced coverage in the LB representative. The corresponding reference interval contains *orf27, orf92*, and *orf93*. This depth-supported interval defines a candidate mitochondrial region but does not establish its exact molecular configuration or biological function.

**Discussion:**

This organellar resource provides a framework for testing evolutionary hypotheses and supporting wolfberry germplasm research.

## 1 Introduction

The genus *Lycium* is a member of the Solanaceae family, with 70 to 80 species (Ragab et al., 2023) occurring in arid and semi-arid regions globally. Among these, *Lycium barbarum* (red wolfberry) (Osman and Awal, 2023) and *L. ruthenicum* (black wolfberry) (Lee and Choi, 2023) are economically and medicinally important due to their abundant bioactive compounds such as carotenoids, polysaccharides, and anthocyanins. Despite their close taxonomic relationship, these species display pronounced phenotypic differences in fruit color and ecological adaptation. Therefore, elucidating the evolutionary patterns underlying this diversification requires a thorough investigation of genetic architecture, with particular emphasis on the organellar genomes, which offer valuable insights into maternal inheritance and lineage divergence.

Plant plastid genomes, or plastomes, typically have a conserved quadripartite structure in which two inverted repeats separate a large single-copy region from a small single-copy region (Qu et al., 2023; Kan et al., 2024a). Within this conserved architecture, codon-usage patterns and small-scale sequence variants can provide information on genetic diversity. Large comparative plastome datasets can also reveal discordant maternal patterns that are consistent with reticulate evolution. However, plastid data alone cannot distinguish hybridization or introgression from incomplete lineage sorting or other processes (Kan et al., 2024b; Wang et al., 2024a).

In contrast to the relatively stable plastome, plant mitochondrial genomes are notable for structural diversity and frequent rearrangement (Palmer and Herbon, 1988; Wang et al., 2024b). Mitogenomes can form branched or multichromosomal configurations mediated by repeated sequences, and rearrangements may generate novel open reading frames (ORFs). However, ORF prediction alone does not establish biological function. Short-read sequencing-depth comparisons can identify candidate interval-scale differences, whereas their exact molecular configurations require targeted or long-read validation. In Lycium, plastomes have been studied more extensively than population-level mitochondrial structural variation.

In this study, we analyzed organellar genomic data from 201 *Lycium* accessions. We used a comparative analysis of this large-scale plastome dataset to characterize genome architecture and maternal genetic structure and to identify patterns compatible with reticulate maternal history. We also assembled one *L. ruthenicum* mitogenome *de novo* and used it as a reference for population-level comparison of the LR and LB complexes. The mitochondrial component focused on interval-scale structural variation identified from sequencing-depth profiles. Candidate ORFs were assessed using structural modeling and transmembrane-helix prediction, with functional interpretation reserved for the Discussion.

## 2 Materials and methods

### 2.1 Material collection, DNA extraction, and sequencing

A total of 201 *Lycium* individuals were collected and taxonomically classified as listed in **Supplementary Table S1**. Genomic DNA was extracted from fresh young leaves from each plant using a modified CTAB protocol (Aboul-Maaty and Oraby, 2019). After quality control, the DNA samples were sequenced at the Beijing Genomics Institute, China. Genomic libraries were prepared using the Illumina TruSeq DNA PCR-Free Library Prep Kit and sequenced on the Illumina HiSeq X Ten platform with a paired-end strategy. Approximately 10 Gb of raw sequencing data were generated per accession. For a conservative theoretical estimate, assuming that 1% of reads were plastid-derived and using a representative plastome length of 155 kb, the expected mean plastome depth was approximately 645×. This value is a theoretical estimate rather than a directly measured per-sample coverage statistic.

### 2.2 Plastome assembly and genome annotation

Complete plastomes for all 201 *Lycium* individuals were assembled *de novo* with GetOrganelle v1.7.7 (Jin et al., 2020). Alternative circularization paths were inspected, and the small single-copy region was oriented consistently after alignment with MAFFT v7.490 (Katoh et al., 2002). Plastomes were annotated with Plastome Genome Annotator v2.0 (Qu et al., 2019), using *L. ruthenicum* accessions OP846050 and MT955897 as annotation references, followed by manual verification of protein-coding start and stop codons. Assembly completeness was additionally checked from the shared gene content: every plastome contained 79 unique CDSs, 30 unique tRNAs, and four unique rRNAs. Duplicated inverted-repeat copies yielded 131 annotated gene copies per plastome (86 CDS, 37 tRNA, and eight rRNA copies). Circular maps were visualized with OGDRAW v1.3.1 (Greiner et al., 2019).

### 2.3 Variant analysis

Snippy v4.6.0 was used to generate an annotated, reference-based catalog of SNVs, small insertions and deletions, and complex variants relative to the A_1Y plastome (**Supplementary Table S2**). Because Snippy was developed primarily for haploid microbial genomes, these calls were used descriptively and were not the primary basis for phylogenetic or selection inference. Complete-plastome and CDS alignments generated independently with MAFFT were used for nucleotide-diversity, phylogenetic, and gene-level analyses as described below. Variant distributions were summarized by genomic region and visualized with ggplot2 (Wickham, 2011).

### 2.4 Nucleotide diversity, selection pressure, and codon usage

All 79 unique plastid CDSs shared across the 201 plastomes were extracted, and this complete 79-CDS set was used for phylogenetic reconstruction. For gene-level sequence-variation analyses, including nucleotide-diversity (π) and substitution-rate estimation, *clpP* and *psbN* were excluded because their marked divergence between the outgroup taxa and *Lycium* compromised the reliability of cross-taxon alignments and estimates; these analyses therefore used the remaining 77 CDSs. Complete plastomes and the 77 retained CDSs were aligned independently with MAFFT v7.490. Nucleotide diversity (π) was calculated with DnaSP v6 (Rozas et al., 2017). Nonsynonymous (dN) and synonymous (dS) substitution rates and dN/dS ratios were estimated with codeml in PAML v4.9 (Gao et al., 2019). Relative synonymous codon usage, effective number of codons, and GC content at third codon positions were calculated with CodonW v1.4 (Sun et al., 2013).

### 2.5 Phylogenetic, population structure, and haplotype-network analyses of the large-scale plastome dataset

The maternal relationships among the 201 *Lycium* accessions were inferred by maximum-likelihood (ML) analysis of a concatenated alignment containing all 79 shared plastid CDSs, using IQ-TREE (Nguyen et al., 2015) with two *Solanum* species as outgroups. The best-fit GTR+I+G4+F substitution model was determined by ModelFinder (Kalyaanamoorthy et al., 2017), and node support was evaluated using 1,000 ultrafast bootstrap replicates. Principal Component Analysis (PCA) was performed with PLINK (Purcell et al., 2007) based on the SNV dataset, while the results were visualized via the OmicShare platform (Mu et al., 2024). Population structure analysis was performed using ADMIXTURE 1.3 (Peter, 2016) with default settings for haploid data using K values from 2 to 10. The optimal K was selected based on the lowest cross-validation (CV) error value across all K values. Haplotypes were identified with DnaSP v6 and used to construct a haplotype network using the median-joining algorithm in PopART v1.7 (Leigh et al., 2015).

### 2.6 Repeat sequence analysis

The MISA web tool (Beier et al., 2017) was used to identify microsatellites across *Lycium* plastomes and 24 additional Solanaceae species, with a threshold of 10 repeats for mononucleotides, six for dinucleotides, and five for tri-, tetra-, penta-, and hexanucleotides. Longer dispersed repetitive elements, including forward, palindromic, reverse, and complement sequences were identified using the REPuter software (Kurtz et al., 2001) with a minimum length of 30 bp and at least 90% sequence identity, allowing for a maximum Hamming distance of 3.

### 2.7 *De novo* assembly and structural visualization of the *Lycium* mitogenome

PacBio high-fidelity long reads from a single *L. ruthenicum* accession (SRR28640618; Xu et al., 2025) were used to assemble one reference mitogenome *de novo* with PMAT v1.5 (Bi et al., 2024). The resulting Graphical Fragment Assembly file was visualized with Bandage v0.8.1 (Wick et al., 2015) to display alternative paths in the reference assembly graph. Separate *de novo* mitogenome assemblies were not generated for all 201 accessions.

### 2.8 Functional annotation and prediction of ORFs in mitogenomes

The *Lycium* reference mitogenome was annotated with PMGA, including protein-coding genes, transfer RNAs, and ribosomal RNAs (Li et al., 2025). Gene boundaries and protein-coding start and stop codons were manually checked, and candidate ORFs were predicted with the GetORFs module in PMGA. Protein structures of candidate ORFs were modeled with AlphaFold 3, and transmembrane helices were evaluated with the TMHMM web server. These in silico predictions were used to assess structural plausibility and were not treated as evidence of biological function. The annotation map was generated with OGDRAW (Lohse et al., 2007).

### 2.9 Population-level mitochondrial structural-variation analysis

Whole-genome sequencing data from 186 accessions representing the LR group (n = 53) and the LB complex (n = 133) were aligned with BWA-MEM to the single *L. ruthenicum* reference mitogenome. The 15 remaining accessions, comprising the *L. chinense*-related LC and LCYN groups (n = 12) and the North American CP group (n = 3), were excluded from this comparison. Separate mitogenomes were not assembled *de novo* for each accession; thus, the comparison was interspecific and population-level. Large structural differences between LR and LB were evaluated from Illumina sequencing-depth profiles generated with PanDepth v2.26 (Yu et al., 2024). Contiguous depth changes were examined across candidate intervals. Only the sequencing-depth analysis was retained for the mitochondrial population-level comparison.

## 3 Results

### 3.1 General features and nucleotide variation in the large-scale wolfberry plastome dataset

The 201 newly assembled *Lycium* plastomes ranged from 154,856 to 155,811 bp and had stable GC contents of 37.8–37.9% (**Figure 1A**). Each assembly displayed the typical quadripartite structure with large and small single-copy regions and two inverted repeats. Each plastome contained 113 unique genes, comprising 79 CDSs, 30 tRNAs, and four rRNAs. After duplicated inverted-repeat copies were counted, each plastome contained 131 annotated gene copies: 86 CDS, 37 tRNA, and eight rRNA copies. The duplicated genes included *rpl2*, *rpl23*, *ycf2*, *ndhB*, *rps7*, *rps12*, *ycf15*, seven tRNAs, and the four rRNA genes.

**Figure 1:**
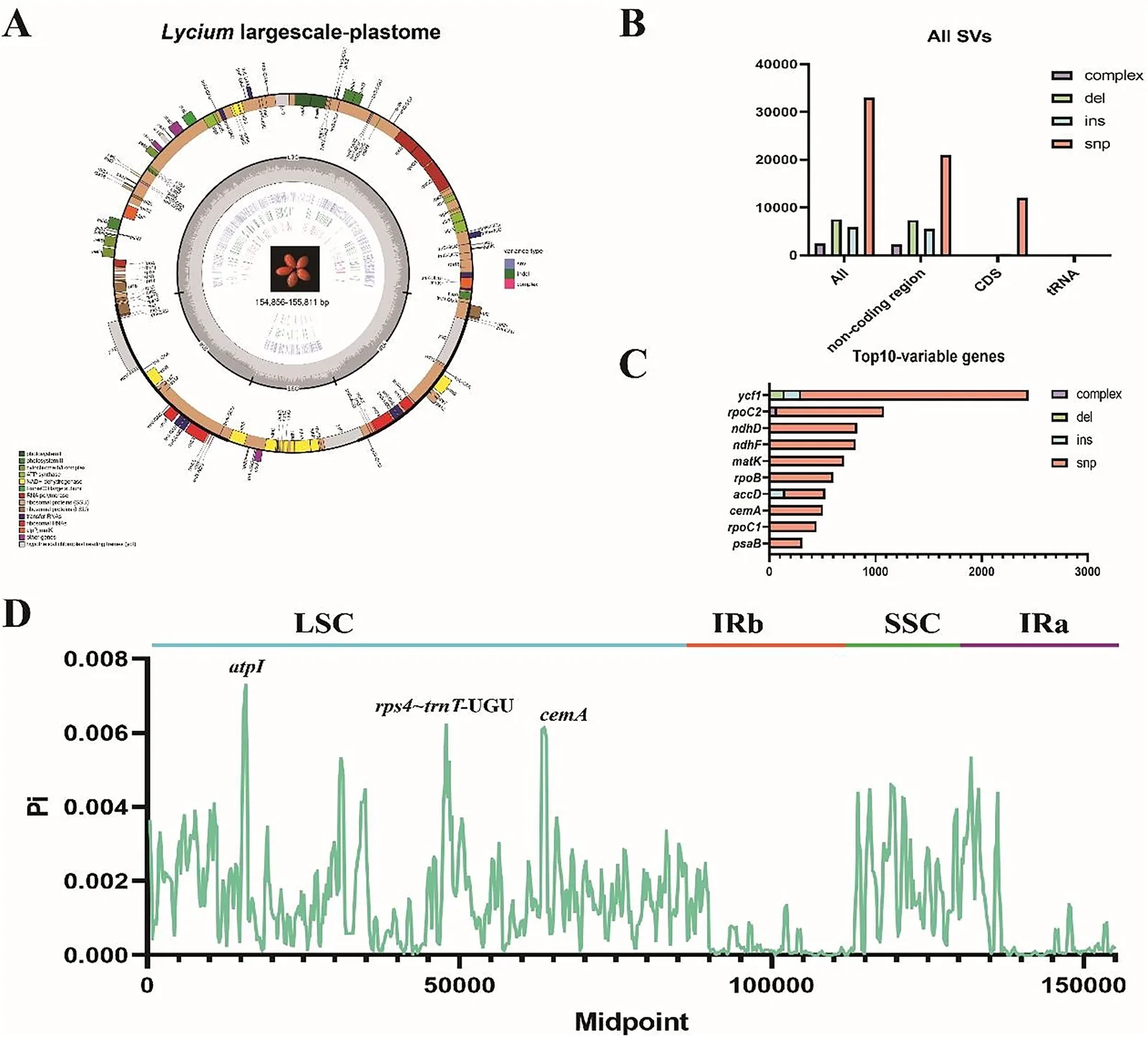
Structural and sequence variation in the large-scale Lycium plastome dataset. **(A)** Annotation map of a representative wolfberry plastome. Genes transcribed clockwise and counterclockwise are shown inside and outside the circle, respectively, and are color-coded by functional class. **(B)** Nucleotide variation across the 201 plastomes. **(C)** The 10 most variable genes among the 77 CDSs retained for gene-level sequence-variation analysis, ranked by variant number. **(D)** Sliding-window nucleotide diversity across the 201 plastomes. The x-axis shows the midpoint of each window, and the y-axis shows nucleotide diversity.

To characterize genetic diversity in the large-scale wolfberry plastome dataset, we aligned the 201 complete plastome sequences. Variants were unevenly distributed across genomic regions (**Figure 1B**). In total, 49,250 variants were detected (**Supplementary Table S2**), comprising 33,296 SNVs, 7,494 deletions, 6,027 insertions, and 2,433 complex variants. SNVs were the most abundant variant type and accounted for approximately 67.6% of all variants. Variant density was higher in the LSC and SSC regions than in the IR regions.

Gene-level sequence-variation analysis of the 77 retained CDSs identified the 10 most variable genes (**Figure 1C**). Among these, *ycf1* was the most variable, followed by *rpoC2* and *ndhD*. Notably, *ycf1* spans the SSC/IR boundary, and the substantial sequence divergence across the 201 samples highlights its potential as a molecular marker for species identification and phylogenetic studies. Other highly variable genes associated with photosynthesis and transcription included *accD*, *ndhF*, and *matK*.

Sliding-window analysis across the 201 plastomes detected nucleotide-diversity (*π*) values from 0 to 0.00728, with a mean of 0.0012 (**Figure 1D**; **Supplementary Table S3**). Nucleotide diversity was lowest in the IRs and highest in the LSC, which contained most mutational hotspots. Hotspots included *atpI*, *rps4-trnT*, and *cemA*.

### 3.2 Maternal relationships and genetic structure within *Lycium*

An ML tree reconstructed from a concatenated alignment of all 79 shared plastid CDSs across the 201 plastomes resolved seven maternal genetic clusters (**Figure 2A**; **Supplementary Table S4**): CP (n = 3), LC (n = 4), LR (n = 53), LBC (n = 53), LCYN (n = 8), LB1 (n = 73), and LB2 (n = 7). LB1, LB2, and LBC were more closely related to one another than to the other clusters. LB1 and LB2 predominantly contained *L. barbarum* accessions, whereas LBC was dominated by *L. barbarum* var. *chinense*. Their proximity in the tree and overlap in the PCA (**Figure 2B**; **Supplementary Table S5**) indicate closely related maternal haplotypes.

**Figure 2:**
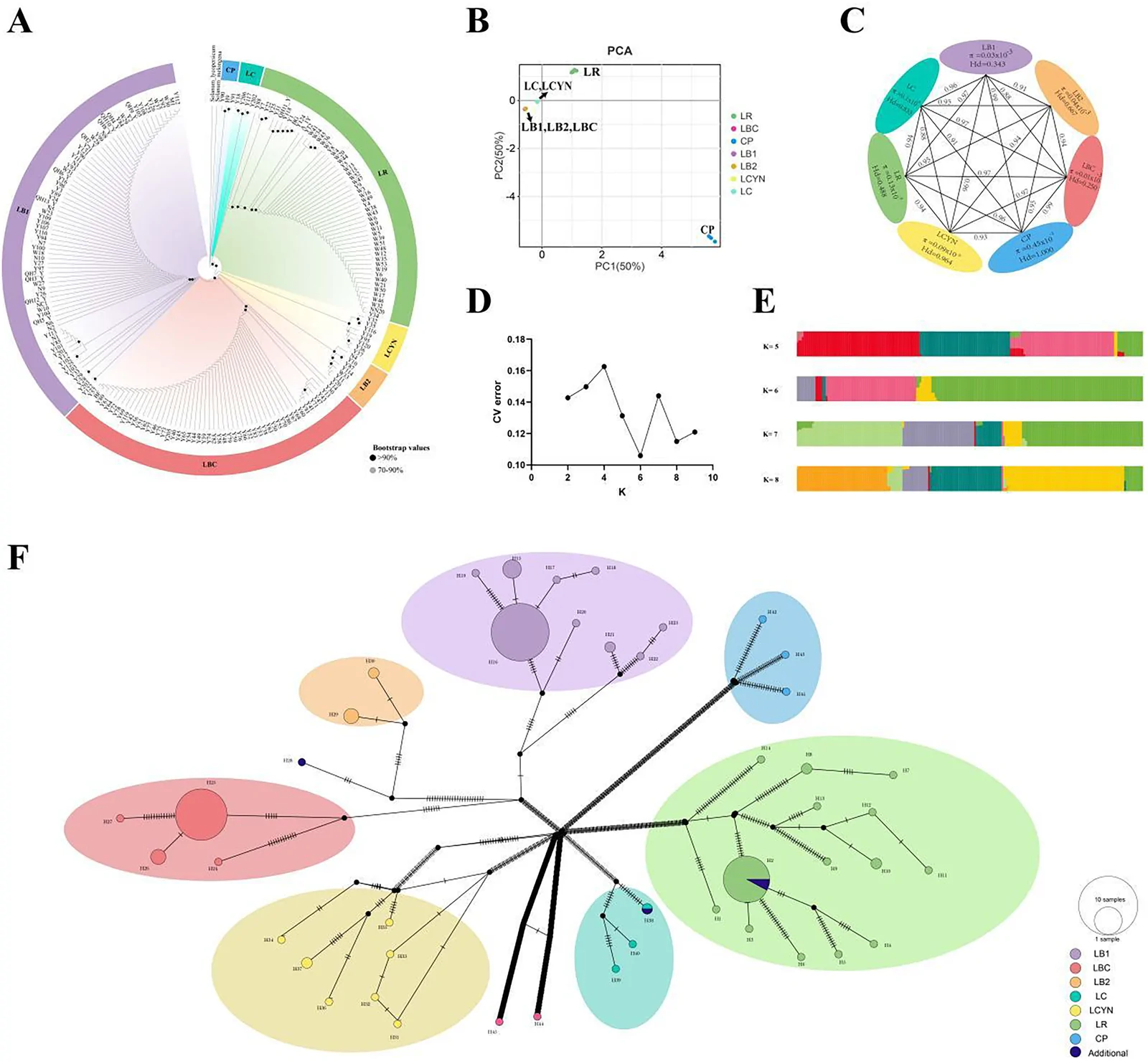
Maternal relationships of plastomes in *Lycium*. **(A)** Maximum-likelihood tree reconstructed from the concatenated alignment of all 79 shared plastid CDSs from 201 wolfberry plastomes. **(B)** Principal component analysis (PCA) showing the first two components. **(C)** Genetic diversity and differentiation among seven genetic clusters of *Lycium*. Numbers above lines connecting two bubbles represent pairwise *Fst* values. **(D)** Cross-validation (CV) errors from K = 2 to K = 9, with the lowest CV error at K = 6. **(E)** Population structure analysis of 201 samples with K values from 5 to 8, showing the optimal K = 6 and the further subdivision at K = 7. **(F)** Haplotype network of wolfberry plastomes, where circle size is proportional to the number of accessions sharing a haplotype.

The LC and LCYN clusters were also closely related in the ML tree and PCA and together represented *L. chinense*-related accessions. The LR cluster, composed predominantly of *L. ruthenicum*, was more distant from the red wolfberry lineages. The CP cluster comprised three North American species, L. exsertum, L. andersonii, and L. berlandieri, and formed the most divergent group. Because CP contained only three accessions, its diversity estimates and geographic interpretation should be treated cautiously. Population-structure analysis showed the lowest cross-validation error at K = 6; K = 7 was additionally displayed to distinguish the LBC maternal cluster for comparison with the ML tree (**Figures 2D, E**; **Supplementary Table S6**).

Pairwise differentiation was highest between CP and LBC (*Fst* = 0.99; **Figure 2C**). The haplotype network resolved 44 haplotypes and supported the close grouping of the LB1-LBC-LB2 complex (**Figure 2F**). Together, the tree, PCA, population-structure analysis, and haplotype network resolved differentiated maternal lineages.

### 3.3 Characterization of repeat sequences and SSR polymorphisms

Repeat sequences, including simple sequence repeats (SSRs) and dispersed repeats, can drive genomic structural variation and are widely utilized as valuable molecular markers. In this study, we characterized SSRs within the *Lycium* genus and across 24 representative *Solanaceae* species. For each group, one representative sample was randomly selected for comparative analysis at the family level of *Solanaceae*.

MISA-web analysis identified three distinct types of SSRs, including mono-, di-, and trinucleotides (**Figure 3A**; **Supplementary Tables S7**, **S8**). Mononucleotide repeats were the most prevalent, accounting for 875 (97.00%) of the total SSRs, followed by dinucleotide repeats (21, 2.33%) and trinucleotide repeats (6, 0.67%). There was a pronounced nucleotide bias, with repeat units containing A/T and AT/AT motifs accounting for 98.89% of all SSRs. These results indicate a strong preference for A/T-rich SSRs in both *Lycium* and wider *Solanaceae* plastomes. The observed bias was consistent across all seven genetic clusters, where mononucleotide SSRs exhibited a substantially higher frequency of A/T types compared to C/G types (**Figure 3B**).

**Figure 3:**
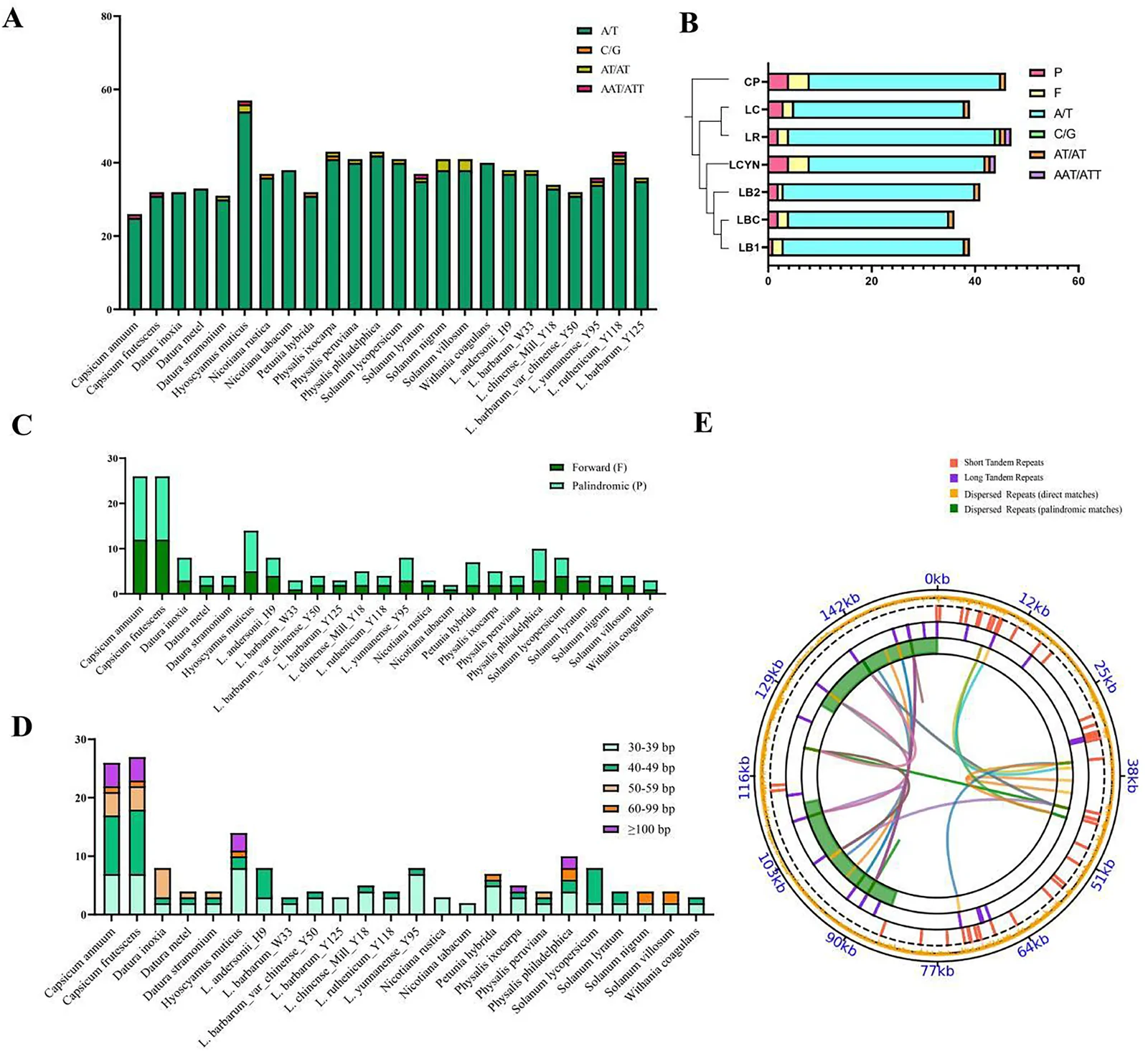
Repeat sequence analysis. **(A)** Number of different simple sequence repeats (SSRs) in Solanaceae plastomes. **(B)** Number of different types of repeats in the seven *Lycium* genetic clusters. **(C)** Type and number of dispersed repeats in Solanaceae plastomes. **(D)** Length distribution of dispersed repeats in Solanaceae plastomes. **(E)** Positions of repeat sequences in the *Lycium* plastome.

The analysis of interspersed repeats at the family level revealed a total of 121 repetitive elements (**Figure 3C**). Of these, palindromic repeats (**Supplementary Table S9**) were the most abundant (55, 45.45%), followed by forward repeats (49, 40.50%), reverse repeats (11, 9.09%), and complementary sequences (6, 4.96%). The lengths of these dispersed repeats varied from 30 to 100 bp, with the 40–49 bp category being the most common among all selected representatives (**Figure 3D**; **Supplementary Table S10**). The genomic distribution of these repeat sequences in *Lycium* species is further illustrated in **Figure 3E**.

### 3.4 Codon usage patterns and selection pressure of plastid genes

The RSCU analysis of *Lycium* protein-coding genes showed a bias toward A/T-ending codons (**Figure 4A**; **Supplementary Table S11**). Among the 64 codons, 30 synonymous codons, most ending in A or U, had RSCU values above 1. Leucine showed the strongest bias for UUA (1.92), followed by alanine for GCU (1.77) and arginine for AGA (1.73). In the ENC-GC3s plot, all genes fell below the standard curve (**Figure 4B**; **Supplementary Table S12**), indicating that mutational bias alone did not explain the observed codon-usage pattern. ENC values ranged from 22.97 for petN to 51.74 for *ycf2*.

**Figure 4:**
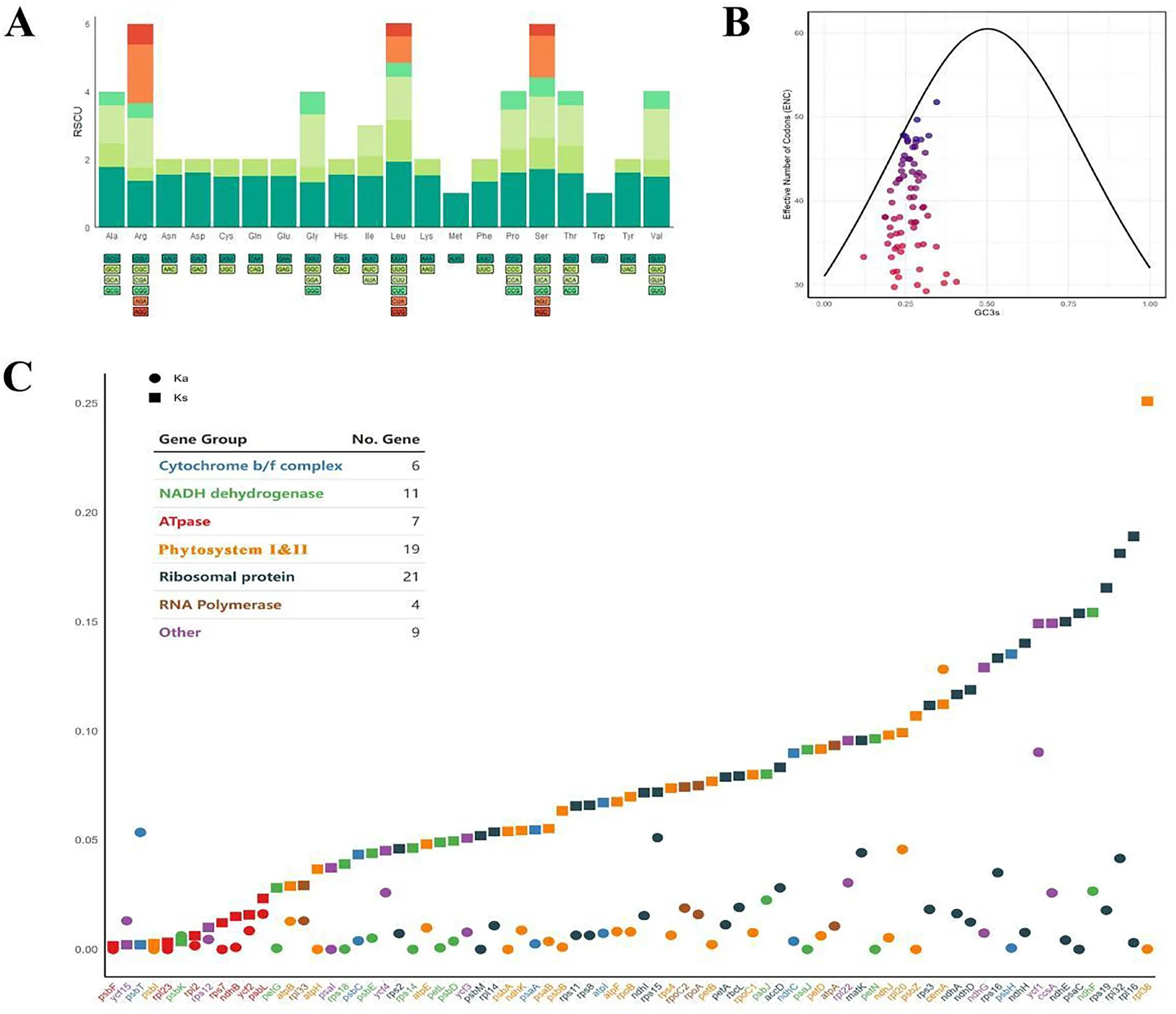
Analysis of codon usage bias and evolutionary rates of the protein-coding genes (PCGs) of *Lycium* plastomes. **(A)** RSCU frequency of 20 amino acids and stop codons, visualized with a color scale representing RSCU values. **(B)** ENC–GC3s plot, where the standard curve indicates the expected effective number of codons (ENC) under mutational pressure alone. **(C)** Nonsynonymous (Ka) and synonymous (Ks) substitution rates for the 77 PCGs retained for gene-level sequence-variation analysis. *clpP* and *psbN* were excluded because their marked divergence between the outgroup taxa and *Lycium* compromised the reliability of cross-taxon estimates.

Substitution rates were estimated from the 77 CDSs retained for gene-level sequence-variation analysis after exclusion of *clpP* and *psbN* (**Figure 4C**; **Supplementary Table S13**). For most genes, dS exceeded dN and dN/dS was below 1, consistent with predominantly purifying selection. The genes *ycf1*, *rpl32*, *rpl36*, *rps19*, and *cemA* showed comparatively elevated substitution rates.

### 3.5 Comparative analysis of *L. ruthenicum* and *L. barbarum* mitogenomes

The single *L. ruthenicum* reference mitogenome was represented by a branched assembly graph with alternative paths (**Figure 5B**), in contrast to the conserved quadripartite plastome architecture. The 402.4-kb reference sequence had a stable GC-content profile and extensive non-coding intergenic regions (**Figure 5A**).

**Figure 5:**
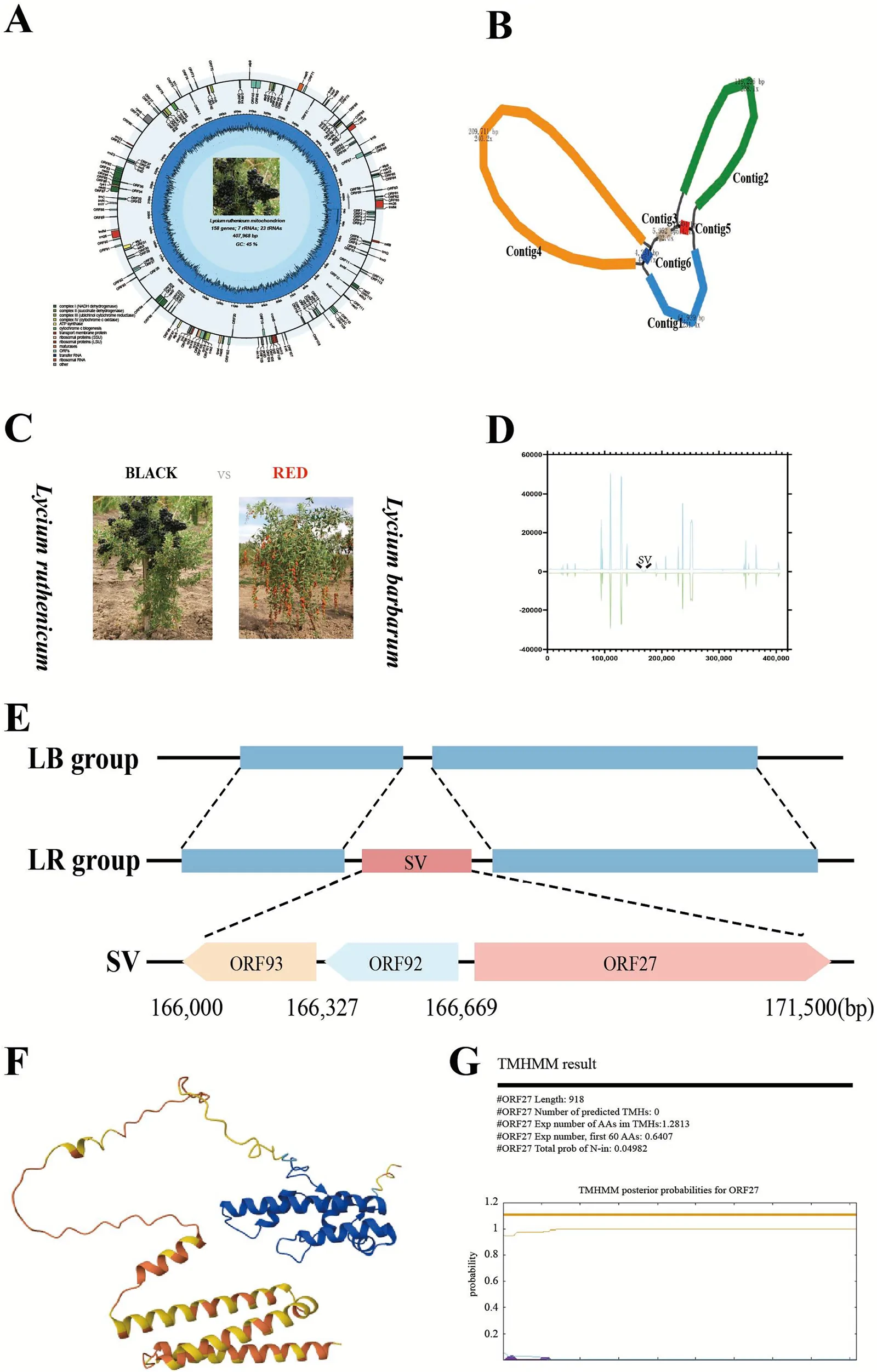
Mitochondrial genome structure and candidate structural variation in *Lycium*. **(A)** Circular annotation map of the *L. ruthenicum* mitogenome. **(B)** Mitogenome structure of an *L. ruthenicum* sample. **(C)** Phenotypic characteristics of *Lycium ruthenicum* (black wolfberry) and *L. barbarum* (red wolfberry). **(D)** Sequencing-depth profiles of LB (Y99) and LR (Y9), highlighting the candidate 5.5-kb interval. **(E)** Annotation of the 5.5-kb reference interval. **(F, G)** AlphaFold 3 structural model and TMHMM analysis of ORF27.

The LR and LB groups exhibited distinct phenotypic characteristics (**Figure 5C**). A candidate mitochondrial structural difference of approximately 5.5 kb between the groups was identified from a contiguous reduction in Illumina read depth generated with PanDepth v2.26 (**Figure 5D**; **Supplementary Table S14**). The corresponding reference segment contained *orf27*, *orf92*, and *orf93* (**Figure 5E**). AlphaFold 3 models and TMHMM predictions indicated that the candidate proteins lacked predicted transmembrane helices (**Figures 5F, G**).

## 4 Discussion

Across the 201 plastomes, genome size (154.8-155.8 kb), GC content, gene order, and the quadripartite architecture were highly conserved. Each plastome contained 79 unique protein-coding genes and 131 annotated gene copies after inverted-repeat duplicates were counted. Nucleotide diversity was lower in the inverted repeats than in the single-copy regions, consistent with patterns reported in other land-plant plastomes (Lado et al., 2020; Loeuille et al., 2021). Dedicated comparative analyses will be required to identify the mechanisms underlying this difference.

Across the 201 plastomes, 33,296 SNVs and more than 13,000 indels provided a detailed map of sequence variation. The high variability of *ycf1*, which spans the SSC/IR boundary, is consistent with its rapid evolution in angiosperms (Huang et al., 2014; Long et al., 2025; Zhu et al., 2016). rpoC2 and ndhD were also among the most variable genes, in agreement with previous observations (Downie and Jansen, 2015; Krawczyk et al., 2022; Xiang et al., 2022). Hotspots in *atpI*, *rps4-trnT*, and *cemA* are useful candidates for marker development. Their possible links to relaxed selection or environmental adaptation remain hypotheses that require functional and population-genomic testing.

The maternal patterns resolved from 201 plastomes show close relatedness within the LB1–LBC–LB2 complex and deeper divergence in LR. The proximity of LB1, LB2, and LBC indicates closely related maternal haplotypes and may reflect recent shared ancestry or reticulate history. Because plastomes represent a single maternally inherited locus, independent nuclear data are required to distinguish hybridization or introgression from incomplete lineage sorting.

The LR plastome cluster was more differentiated from the red-wolfberry clusters, consistent with its distinct placement in previous genomic analyses (Cao et al., 2021). However, a plastome topology alone cannot date divergence or establish a unique evolutionary trajectory. Broader taxon sampling and nuclear-genomic evidence are needed to test the timing and causes of the observed maternal differentiation.

The CP cluster comprised *L. exsertum*, *L. andersonii*, and *L. berlandieri*, three North American species represented by one accession each. Its divergent position is therefore best interpreted as a geographically and taxonomically distinct maternal lineage in this dataset, not as a Eurasian cluster. This interpretation remains provisional because the cluster contains only three accessions.

Repeat analysis across the large-scale *Lycium* plastome dataset and representative Solanaceae species showed strong structural conservation. Mononucleotide A/T repeats accounted for 97% of SSRs, consistent with the A/T-rich repeat profiles reported in land-plant plastomes (Jiang et al., 2024; Abdullah et al., 2021). Repeat profiles were generally conserved across the seven genetic clusters, including LR and the cultivated LB clusters. The identified SSRs and dispersed repeats may therefore provide markers for distinguishing maternal genetic groups. The prevalence of palindromic and forward repeats further characterized repeat diversity across the dataset.

Codon-usage patterns in *Lycium* plastomes likely reflect contributions from both mutational bias and selection. The preference for A/T-ending codons is common in land-plant plastomes and may influence translation efficiency (Zhang et al., 2022; Yengkhom et al., 2019; Chakraborty et al., 2020; Peng et al., 2023). Most genes had low dN/dS ratios, consistent with widespread purifying selection. *ycf1* and *cemA* showed comparatively high substitution rates, as reported in other angiosperms (Wu et al., 2020; Wen et al., 2021; Fan et al., 2025). However, these rate differences remain descriptive and do not establish positive selection. *clpP* and *psbN* were excluded only from the gene-level sequence-variation analyses because their marked divergence between the outgroup taxa and *Lycium* compromised cross-taxon estimates; both genes were retained in the 79-CDS phylogenetic alignment.

In contrast to the conserved plastome organization, the single L. ruthenicum reference mitogenome was represented by a branched assembly graph with alternative paths. This topology is compatible with repeat-associated recombination documented in plant mitochondria (Gualberto and Newton, 2017), but the present data do not quantify recombination frequency or establish that every path represents a stable *in vivo* molecule. Long-read mapping and molecule-level validation will be needed to resolve the relative abundance and biological status of alternative mitochondrial conformations.

Illumina depth profiling identified a candidate 5.5-kb interval through a contiguous reduction in coverage in the LB representative relative to *L. ruthenicum*. This pattern supports an interval-scale structural difference but does not establish its exact molecular configuration or functional effect. Independent long-read assemblies and targeted validation across LR and LB accessions will be required to resolve the structure of this interval.

The 5.5-kb reference region contains *orf27*, *orf92*, and *orf93*. AlphaFold 3 and TMHMM analyses provide candidate structural models and indicate no predicted transmembrane helices. These predictions do not establish that the ORFs are expressed, translated, or physiologically active. Functional analyses will be required to determine whether these ORFs have biological activity, while comparative sampling across *Lycium* will be needed to reconstruct their evolutionary histories.

In summary, plastome analyses resolved differentiated maternal lineages within *Lycium*, and mitochondrial depth profiles identified a candidate 5.5-kb interval-scale difference between LR and LB. This candidate region provides a focused target for structural and functional validation.

## 5 Conclusions

This study provides a comparative organellar resource for 201 *Lycium* accessions. The plastome data resolved seven maternal genetic clusters and identified conserved genome architecture alongside lineage-informative sequence variation. Depth-based comparison with a single *L. ruthenicum* reference mitogenome identified a candidate 5.5-kb structural difference between LR and LB. This depth-supported interval is a candidate region for mitochondrial difference in this genus. Future work should investigate the biological functions of the candidate ORFs and examine their evolutionary histories across *Lycium*.

## Data Availability

The raw sequencing data presented in the study are deposited in the NCBI repository, accession number PRJNA1537283. Derived datasets, including annotations for the large-scale plastome dataset and the L. ruthenicum reference mitogenome annotation, are deposited in the Figshare repository (https://doi.org/10.6084/m9.figshare.31475467). The complete **Supplementary Tables** accompany the revised submission.
